# Research on computing task scheduling method for distributed heterogeneous parallel systems

**DOI:** 10.1038/s41598-025-94068-0

**Published:** 2025-03-15

**Authors:** Xianzhi Cao, Chong Chen, Shiwei Li, Chang Lv, Jiali Li, Jian Wang

**Affiliations:** College of Computer and Control Engineering, Northeast Forestry University, Harbin, 150040 China

**Keywords:** Heterogeneous parallel, Dynamic scheduling, Directed acyclic graph, Dynamic redundancy, Mathematics and computing, Computational science

## Abstract

With the explosive growth of terminal devices, scheduling massive parallel task streams has become a core challenge for distributed platforms. For computing resource providers, enhancing reliability, shortening response times, and reducing costs are significant challenges, particularly in achieving energy efficiency through scheduling to realize green computing. This paper investigates the heterogeneous parallel task flow scheduling problem to minimize system energy consumption under response time constraints. First, for a set of independent tasks capable of parallel computation on heterogeneous terminals, the task scheduling is performed according to the computational resource capabilities of each terminal. The problem is modeled as a mixed-integer nonlinear programming problem using a Directed Acyclic Graph as the input model. Then, a dynamic scheduling method based on heuristic and reinforcement learning algorithms is proposed to schedule the task flows. Furthermore, dynamic redundancy is applied to certain tasks based on reliability analysis to enhance system fault tolerance and improve service quality. Experimental results show that our method can achieve significant improvements, reducing energy consumption by 14.3% compared to existing approaches on two practical workflow instances.

## Introduction

With the development of information technology, the rapid increase in the number of terminal devices in networks has not only led to an exponential growth in data volume but also significantly increased the consumption of communication resources^1^. In the face of increasingly stringent real-time requirements in application scenarios, Mobile Edge Computing (MEC) has emerged as a new computing paradigm. Offloading computational tasks to the network edge enables local data processing and real-time responses, which has attracted widespread attention^2,3^. However, the rise of MEC has introduced new challenges, especially in the context of heterogeneous edge environments^4,5^. Terminal devices in these environments, such as CPUs, GPUs, FPGAs, and base stations, exhibit significant variations in computational capabilities and communication quality, making resource allocation and task scheduling particularly complex^6^. Meanwhile, due to the increasing complexity of tasks and growing computational demands, complex computational tasks are often decomposed into several subtasks, forming workflow tasks with dependencies^7^. A well-designed workflow task scheduling can better utilize the computational capabilities of multi-core processors, significantly improve resource utilization and task execution efficiency, and effectively reduce system energy consumption, enhancing task completion reliability^8–10^.

As the scheduling problem is fundamentally an NP (Non-deterministic Polynomial) hard problem^11^, previous methods often rely on heuristic algorithms to solve it^12^. Scheduling technology occupies a critical position in distributed computing systems, and many scheduling techniques have been proposed before, focusing on optimizing various performance metrics such as response time, reliability, energy consumption, quality of service, cloud throughput, and resource utilization^13,14^. However, most scheduling methods focus on only one or two metrics, failing to balance multiple performance requirements comprehensively^15,16^. For example, some studies focus on how to meet task response time requirements^17^. Many scholars have also proposed solutions to the reliability problem^18^. However, in practical engineering, it is necessary to balance conflicting metrics such as response time, reliability, and energy consumption^6,19,20^.

The research motivation behind this paper is to address the gap in existing scheduling strategies by proposing a novel, unified approach that balances multiple performance metrics-such as task execution time, reliability, and energy consumption-while accounting for the heterogeneity of edge resources^21,22^. Unlike traditional approaches that optimize individual metrics in isolation, our approach aims to provide a holistic solution that adapts to the specific needs of heterogeneous edge environments. In particular, we focus on task decomposition and workflow scheduling in MEC, where tasks often have complex dependencies and need to be allocated across different processing units to minimize energy consumption and meet real-time constraints. At the same time, the fault tolerance of the dispatching system is considered.

By addressing these challenges, this paper aims to contribute to advancing task scheduling algorithms that are both efficient and scalable for real-world edge computing scenarios, providing a foundation for optimizing system performance across a wide range of heterogeneous devices.

The main contributions of this paper can be summarized as follows:

1. Given the heterogeneous parallelism of the system, we use a DAG as the input and analyze in detail the difference in computing time and energy consumption per unit time of each task on different processors. By quantifying computing resources, the dependencies between subtasks, as well as potential combinations and solutions on heterogeneous devices, are clearly demonstrated.
2. Considering the competition for computational and communication resources in heterogeneous terminals, we propose the HRLHS (Heuristic and Reinforcement Learning-based Heterogeneous Scheduling) algorithm. This algorithm combines the strengths of Particle Swarm Optimization (PSO) and Reinforcement Learning (RL) techniques, and is enhanced through various improvements to achieve efficient green task scheduling, minimizing system energy consumption.
3. To enhance the reliability of scheduling results and overall system performance, we incorporate a dynamic redundancy mechanism in our scheduling framework. This mechanism performs backup tasks for those with lower reliability, thereby improving system fault tolerance and robustness.
4. The proposed algorithm is experimentally evaluated on two practical workflow cases. The experimental results demonstrate that, compared to existing methods, the proposed approach not only exhibits excellent performance in energy-efficient task scheduling but also achieves the expected targets for response rate and response time.

The remainder of this paper is organized as follows. The Sect. Related works describes related works. The Sect. Problem statement provides the system model and problem description. The Sect. Energy-saving scheduling algorithm presents the dynamic scheduling algorithm based on heuristic and reinforcement learning for minimizing energy consumption in complex computational resources. The Sect. Experiments and discussion demonstrates the experiments and results. Finally, the Sect. Conclusion concludes the paper.

## Related works

This section reviews the latest research on task scheduling methods^23,24^. We then analyzed the objectives, strengths, weaknesses, and methods of each study through a table. Xu et al. divided tasks into stages based on data dependencies and dynamically evaluated node performance for task classification, optimizing resource allocation to minimize task completion time and reduce the scheduling length ratio. However, its applicability is limited to scenarios with clearly defined task dependencies and may face challenges in handling highly dynamic or complex task relationships^25^. Li et al. proposed a Q-learning-based scheduling algorithm for adaptive resource management in complex computing environments. Their approach demonstrates advanced task completion time and resource utilization. However, Q-learning’s performance may be influenced by the choice of state and action representations and may require substantial time to converge in highly dynamic environments^26^. Liao et al. proposed a binary-coded genetic algorithm for adaptive offloading in ultra-dense cellular networks, suitable for low-latency and low-energy scenarios, but may underperform in complex applications^27^. Hu et al. optimized the joint problem of request offloading and resource scheduling. The goal was to minimize task response times, demonstrating good performance in ultra-dense edge computing environments^28^. Tuli et al. proposed a real-time scheduling scheme based on the A3C algorithm and achieved distributed learning with multiple agents through the R2N2 framework. This approach adapts by adjusting hyperparameters to optimize scheduling decisions. However, the method is limited by its focus on a fixed number of edge nodes and tasks, which constrains its applicability^29^. Mahesar et al. proposed an improved scheduling strategy, aiming to minimize workflow execution costs while meeting user-specified deadlines and reliability requirements^30^.

However, the above study does not fully consider fault tolerance when discussing scheduling strategies, which ensures that the system remains up and running even in the event of a failure of the processor, communication link, or other components, thus providing high availability and mitigating the damage caused by the failure^31^. Dai proposed a Q-learning-based dynamic fault-tolerant scheduling algorithm for microservice orchestration, improving system stability and resource allocation. It uses Kubernetes and LSTM (Long Short-Term Memory) networks for fault prediction. While the approach enhances service availability and recovery time, it faces limitations in simulated environments and fault type coverage^32^. Long et al. adopted a primary-backup strategy to tolerate task failures in edge nodes and combined deep reinforcement learning to optimize task scheduling, significantly improving task completion rates^33^. Yao et al. combined resubmission and replication to improve fault tolerance for independent tasks with deadlines in cloud systems, selecting strategies and reserving resources based on task and cloud resource characteristics^34^. Yin et al. developed a genetic algorithm-based fault-tolerant allocation strategy, significantly improving task completion time, resource utilization, and reliability^35^. Chawla et al. employed replication and migration techniques for fault-tolerant scheduling, minimizing processors, and balancing the load to optimize resource utilization and reduce losses from resource failures^36^. However, excessive redundancy, re-submission, task replication, and migration strategies, while contributing to improved system robustness, may lead to energy waste, ultimately affecting resource efficiency. Therefore, balancing fault tolerance and resource utilization efficiency represents a key challenge in practical applications. Table 1 shows the classification of the scheduling approaches with respect to the algorithms, metrics, advantages, and disadvantages.

**Table 1:** Classification of the scheduling algorithms.

References	Alg	Response time	Energy consumption	Reliability	Advantages	Disadvantages
Xu et al.^25^	GATS-TS	$\checkmark$	✗	✗	Enhanced Resource Utilization Adaptive Performance Evaluation	High Computational Complexity Dependency on Task Classification
Li et al.^26^	Q-learning	$\checkmark$	$\checkmark$	✗	Dynamic Adaptability Improved Resource Allocation	Complexity in Implementation Dependence on Feedback
Liao et al.^27^	GA	$\checkmark$	$\checkmark$	✗	Suitability for Ultra-Dense 5G Adaptive Offloading Decision	Computational Complexity Dependency on Parameter Selection
Hu et al.^28^	NCGG NSGA	$\checkmark$	$\checkmark$	✗	Balance Multiple Goals Rapid Resource Allocation	Unable to Face Failures Effectively Large Computational Overhead
Tuli et al.^29^	A3C R2N2	$\checkmark$	$\checkmark$	✗	Negligible Scheduling Overhead Adaptive Dynamic Environments	High Computational Cost Complexity in Implementation
Mahesar et al.^30^	RWSMS	$\checkmark$	$\checkmark$	$\checkmark$	Effective for Real-world Workflow	No Guarantee of Optimal Solution Rely on Resource Allocation
Dai et al.^32^	Q-learning	$\checkmark$	✗	$\checkmark$	Improved System Stability Fault Prediction Capability	Inadequate Coverage of Fault Types Lack of Adaptability
Long et al.^33^	DQN	✗	✗	$\checkmark$	Efficient Task Scheduling Improving Task Completion Rates	Depending on Environment Setup Not Suitable for Complex Workflows
Yao et al.^34^	HFTSA	$\checkmark$	✗	$\checkmark$	Dynamic Resource Adjustment Efficient Fault-tolerant Strategy	Impact of Cloud Resource Performance Fluctuations
Yin et al.^35^	NSGA-III	$\checkmark$	$\checkmark$	$\checkmark$	Improved Performance on All Three Objectives	Over-reliance on Initial Population Selection.
Chawla et al.^36^	Heuristics	$\checkmark$	✗	$\checkmark$	Enhanced Load Balance Combination of Multiple Metrics	Overhead from Replication Increased Algorithmic Complexity
our research	HRLHS (PSO&RL)	$\checkmark$	$\checkmark$	$\checkmark$	Efficient Green Task Scheduling Improved System Fault Tolerance Expected Response Time Targets	–

## Problem statement

**Fig. 1:**
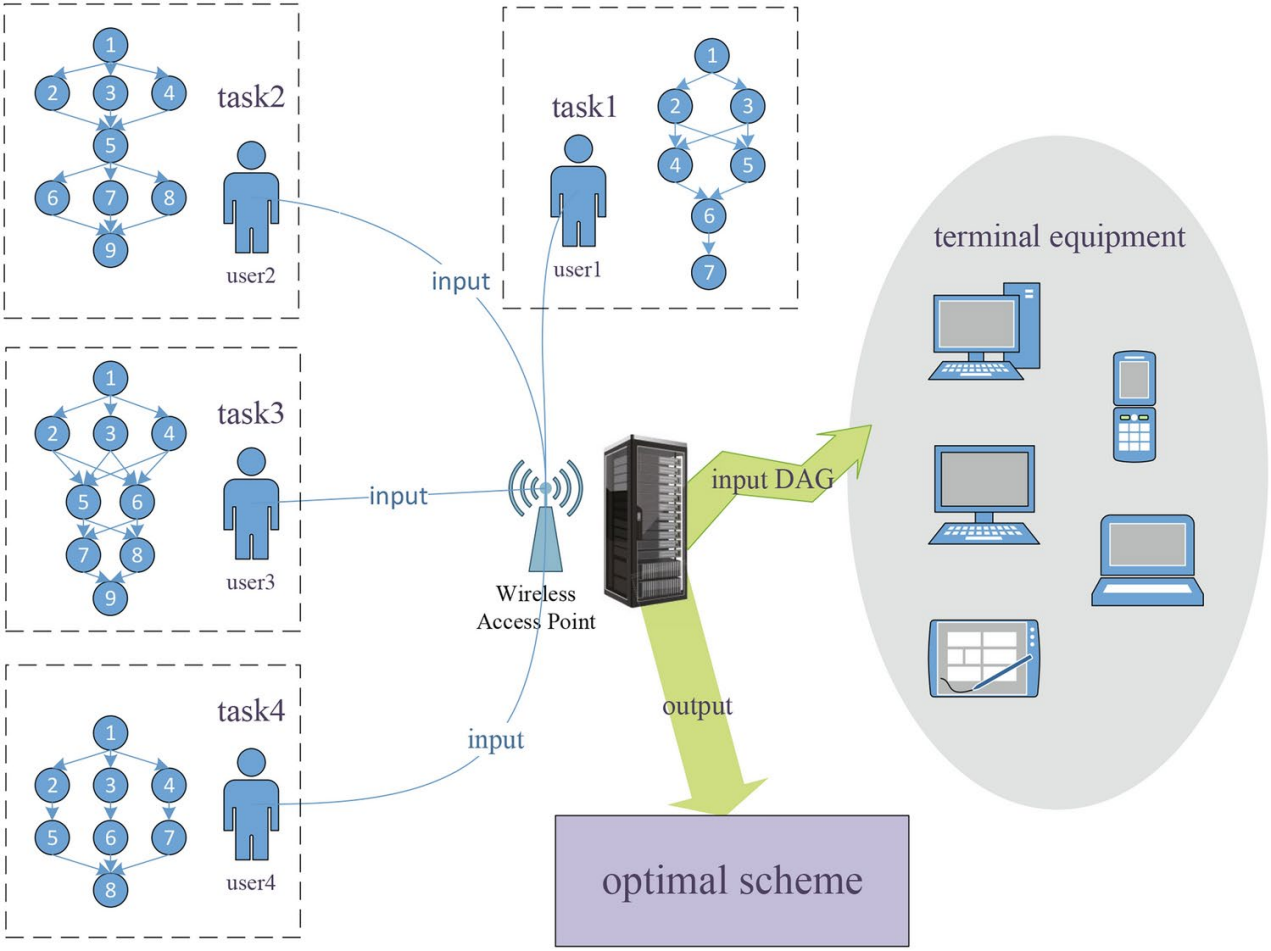
Task scheduling system framework (This figure is original and created by the author.).

To address the effective utilization of complex computing resources in distributed systems, as shown in Fig. 1, large model tasks that can be processed in parallel are first gathered into a central scheduler. The input task set is represented by a DAG model, which clearly depicts the dependencies between workflow tasks. Subsequently, this paper proposes an efficient scheduling strategy aimed at optimizing the utilization of computing resources through parallel processing across multiple heterogeneous terminal processors, thereby reducing overall processing time and energy consumption. The important symbols used in this section are defined in Table 2.

**Table 2:** Main notations in this study.

Notation	Description
*D*	DAG Workflow
$T_i$	The *i*-th workflow computational task
$C_j$	The *j*-th terminal processor
$E_{ij}$	The propagation delay between the *i*-th task and the j-th task
$A_{ij}$	The processing delay of the *j*-th task on the *i*-th processor
*M*	Number of processors
*N*	Number of tasks
$O_i$	Current task
$pr_i$	The predecessor task of the *i*-th task
$su_i$	The successor task of the *i*-th task
$tm_{sta}^{o(i)}$	The start time of the *i*-th task
$tm_{end}^{o(i)}$	The end time of the *i*-th task
$p_{sta}^{m(i)}$	The static energy consumption of the *i*-th processor
$p_{dyn}^{m(i)}$	The dynamic energy consumption of the *i*-th processor
$h_i$	Processor state
*ms*	The maximum time span of the workflow tasks
$\lambda _j$	The reliability per unit time of the *j*-th processor
$R_D$	The total reliability of the workflow tasks
$R_j^i$	The reliability of the *i*-th task running on the *j*-th processor
$x_{ij}$	The assignment of the *i*-th task on the *j*-th processor

### System model

A practical distributed platform is equipped with a large number of complex computational resources. Let $C=\{C_1,C_2,\ldots C_M \}$ represents the *M* parallel heterogeneous terminals, and let $T=\{T_1,T_2,\ldots T_N \}$ represents the *N* tasks. When these *N* tasks need to be scheduled across these *M* processors, the actual parallel workflow can be represented by a DAG *D*(*E*, *A*, *P*), where *E* denotes the task propagation matrix^37^, which indicates the dependency relationships between tasks and the communication delays, *A* represents the task execution time matrix, and *P* is the power consumption matrix. These heterogeneous terminals are interconnected via communication links, and the data transmission rates between processors differ. The time to process the same task $W_{ij}$ and the communication delay $E_ij$ for propagating the same task also vary depending on the processors, thus simulating the real-world scenario of heterogeneous terminals.

**Fig. 2:**
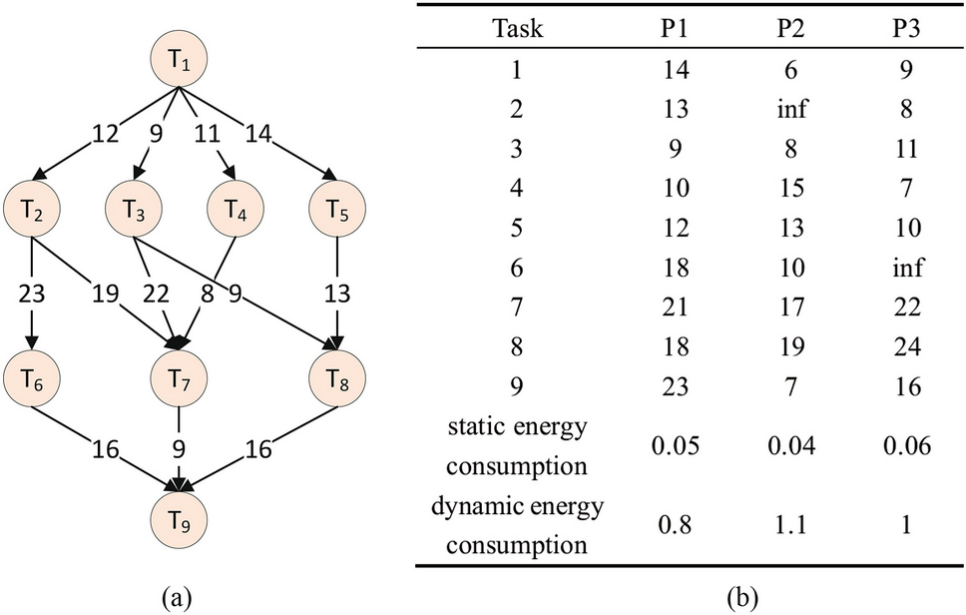
An example of a DAG (**a**), along with the time and energy consumption of the task on a heterogeneous processor (**b**).

Figure 2 illustrates an example of a DAG model, where nine tasks are executed on three different processors. In this DAG model, each node represents a task, and each edge represents the dependency relationship between tasks and the communication delay. If there is an edge from task $T_i$ to task $T_j$,$T_i$ is considered the predecessor task of $T_j$, and $T_j$ is the successor task of $T_i$. The edge indicates that the successor task must start execution only after the completion of its predecessor task. Suppose the current task is $O_i$, then its predecessors $pr_i$ and successors $su_i$ can be found within the DAG. A task can have multiple predecessors and successors, and $O_i$ must execute after all of its predecessors $pr_i$ are completed. For two tasks with precedence relationships, one task’s input depends on the output of another task. Therefore, when these two tasks execute on different processors, both the propagation delay and the execution delay need to be considered. However, if both tasks are executed on the same processor, only the execution delay is relevant. Due to task heterogeneity, even on the same processor, execution delays may vary. Similarly, due to the heterogeneity of the terminals and the varying computational capabilities of the processors, different processors require different execution times for the same task. If a processor lacks the capability to execute a specific task, the execution time is considered infinite. In the DAG, a task without any predecessor is called an entry task, and a task without any successor is called an exit task. If the DAG has multiple entry or exit tasks, a zero-weight virtual entry or exit task can be added to ensure the system has only one entry and one exit task. The first task is denoted as the entry task, and the last task is designated as the exit task, i.e., $T_1$ is the entry task, and $T_N$ is the exit task. It is assumed that all tasks share the same deadline and are executed in a non-preemptive manner.

### Energy consumption model

The total energy consumption is defined as the sum of dynamic energy consumption and static energy consumption^19^, which can be mathematically expressed as follows.

1 $$\begin{aligned} W_{all}=W_{sta}+W_{dyn}=\sum _{i=1}^{M} w_{{sta}}^i + \sum _{i=1}^{M} w_{{dyn}}^i \end{aligned}$$

where a constant frequency $f_i$ is set for each processor, and for the convenience of calculation, the maximum frequency $f_{max}$ is normalized to 1, so $f_i$ satisfy $f_i\in (0,1)$. The dynamic energy consumption for each task during execution is given by:

2 $$\begin{aligned} W_{dyn} = \sum _{i=1}^{N} P_{dyn}^{m(i)} \cdot f_i \cdot A_{ij} \end{aligned}$$

Due to the heterogeneity of complex computational resources, processors with better performance tend to have shorter processing times and higher power consumption when handling the same task. Therefore, for a given task, the product of processing time and power consumption represents the energy consumed. However, in reality, the choice of processor and the task execution order are not the only influencing factors. A flag *h* is introduced to represent the processor’s switch status: if the processor is running, $h=1$; otherwise, $h=0$. Here, the static energy consumption is defined as:

3 $$\begin{aligned} W_{sta} = \sum _{i=1}^{M} P_{sta}^{m(i)} \cdot h_i \cdot ms \end{aligned}$$

It can be observed that the minimum energy consumption for each individual task does not necessarily guarantee the overall minimum energy consumption. This is because a reduction in the dynamic energy consumption of a single task may result in an increase in static energy consumption. Specifically, the reduction in energy consumption might come at the cost of increased response time, and this additional static energy consumption due to the response time must be shared by all processors. Therefore, it is possible to strategically choose and turn off certain processors to reduce overall energy consumption. For task $O_i$, which must execute after all its predecessors $pr_i$ are completed, assuming it has only one predecessor if both tasks are assigned to the same processor, then the following holds:

4 $$\begin{aligned} tm_{sta}^{o(i)} = tm_{end}^{pr(i)} \end{aligned}$$

If the current task and its predecessor are assigned to different processors, then the following holds:

5 $$\begin{aligned} tm_{sta}^{o(i)} = tm_{end}^{pr(i)} + E_{o(i)pr(i)} \end{aligned}$$

Therefore, when selecting a processor, one must consider not only the dynamic energy consumption required by the task itself, but also whether task migration is necessary, which may incur additional response time loss. Furthermore, the choice of the current task’s state will affect the selection of its successor tasks.

### Failure model

For a real system, the reliability probability of each processor is unstable. The system’s reliability is defined as the probability that the system operates continuously without failure over a given time interval, and the probability of failure follows a Poisson distribution^38^. Therefore, the reliability is expressed as $e^{-\lambda \Delta }$, where $\Delta$ is a small time interval and $\lambda$ is the constant failure rate of the processor per unit time. For a specific task $T_i$, the probability that it runs on processor $C_j$ without failure is given by:

6 $$\begin{aligned} R_j^i = e^{-\lambda _j A_{ji}} \end{aligned}$$

For each processor, its reliability parameter is also influenced by its frequency. Assuming the frequency of the processor is $f_j$, the reliability can be expressed as:

7 $$\begin{aligned} \lambda _j = \lambda _{j,{max}} \times 10^{\frac{d(f_{j,{max}} - f_j)}{f_{j,{max}} - f_{j,{min}}}} \end{aligned}$$

where d is a constant representing the sensitivity of the failure rate to frequency scaling. Therefore, equation (6) can be computed as:

8 $$\begin{aligned} R_j^i = e^{-A_{ji} \times \lambda _{j,max} \times 10^{\frac{d(f_{j,max} - f_j)}{f_{j,max} - f_{j,min}}}} \end{aligned}$$

which illustrates that the reliability decreases monotonically as the frequency decreases.

Failures can result from processor malfunctions or incorrect task execution. In this study, heartbeat detection technology is proposed, where the unit time is set as the shortest execution time of a task. Periodic signals are sent to confirm whether the processor nodes in the system are operating normally. If the expected heartbeat signal is not received within the specified time, the system assumes that the corresponding node may have failed and will take appropriate actions, such as re-executing the task, switching to a backup node, or issuing a warning. Therefore, it can be assumed that the failure of a single task will not affect the processor’s ability to handle other tasks in the next time period. Thus, the probability that the entire workflow task does not experience any failures is given by:

9 $$\begin{aligned} R_D = \prod _{T_i \in D} R_j^i \end{aligned}$$

where $R_j^i$ represents the reliability of the *i*-th task being scheduled on the *j*-th processor during the scheduling process.

### Problem formulations and constraints

Given a real-time parallel workflow *D*, the problem at hand is to select a scheduling algorithm that minimizes energy consumption while satisfying the response time and reliability requirements. Based on the above conclusions, the following constraints can be derived:

10 $$\begin{aligned} & \sum _{j=1}^{M} x_{i,j} = 1, \quad \forall i = 1, \dots , N \end{aligned}$$

11 $$\begin{aligned} & tm_{end}^{o(i)} \ge \max \left( tm_{end}^{p(i)} + \left( h_{o(i)} \oplus h_{p(i)} \right) \times E_{o(i)p(i)} + A_{ij} \times h_i \right) \end{aligned}$$

12 $$\begin{aligned} & \begin{aligned} \left( \sum _{j=1}^{M} x_{i1,j} \times x_{i2,j} \right) \cdot \left( tm_{sta}^{i1} + \sum _{j=1}^{M} x_{i1,j} \times A_{i1,j} \times \frac{1}{f_{i1}} - tm_{sta}^{i2} \right) \cdot \\ \left( tm_{sta}^{i2} + \sum _{j=1}^{M} x_{i2,j} \times A_{i2,j} \times \frac{1}{f_{i2}} - tm_{sta}^{i1} \right) \le 0 \end{aligned} \end{aligned}$$

13 $$\begin{aligned} & tm_{end}^{o(i)} \le tm_{end}^{o(N)} \end{aligned}$$

The constraint (10) indicates that each task can only be executed on exactly one processor. Constraint (11) ensures that task dependencies are respected, meaning that a task can only be executed after its predecessor task has been completed. Constraint (12) prohibits task preemption. Constraint (13) specifies that the completion time of each computational task must be before the overall deadline.

From the above equations and the energy consumption model, it can be observed that the nonlinear calculations caused by task dependencies and the nonlinear multiplicative relationship between communication energy consumption and task allocation lead to a nonlinear objective function for the scheduling problem. Furthermore, based on the definition of variables, it is evident that the processor switch state is a binary variable, while the task completion time is a continuous variable. As this problem contains both integer and continuous variables, it is classified as Mixed-Integer Nonlinear Programming (NLMIP) problem. The complexity of this problem primarily arises from the following aspects:

1. Task Dependencies: The task dependencies require ensuring that a successor task can only begin after all its predecessor tasks have been completed. This dependency introduces complex communication delay calculations and scheduling decisions in multi-processor systems.
2. Multi-Processor Scheduling: Assigning multiple tasks to different processors while minimizing the total energy consumption or completion time is a classic combinatorial optimization problem.
3. Nonlinear Objective Function: The energy consumption optimization problem is typically nonlinear, especially when energy consumption is related to processor load, communication time, and other factors.

Therefore, this problem can be classified as an NP-hard problem, and in this paper, an optimization algorithm is proposed to solve it.

## Energy-saving scheduling algorithm

**Fig. 3:**
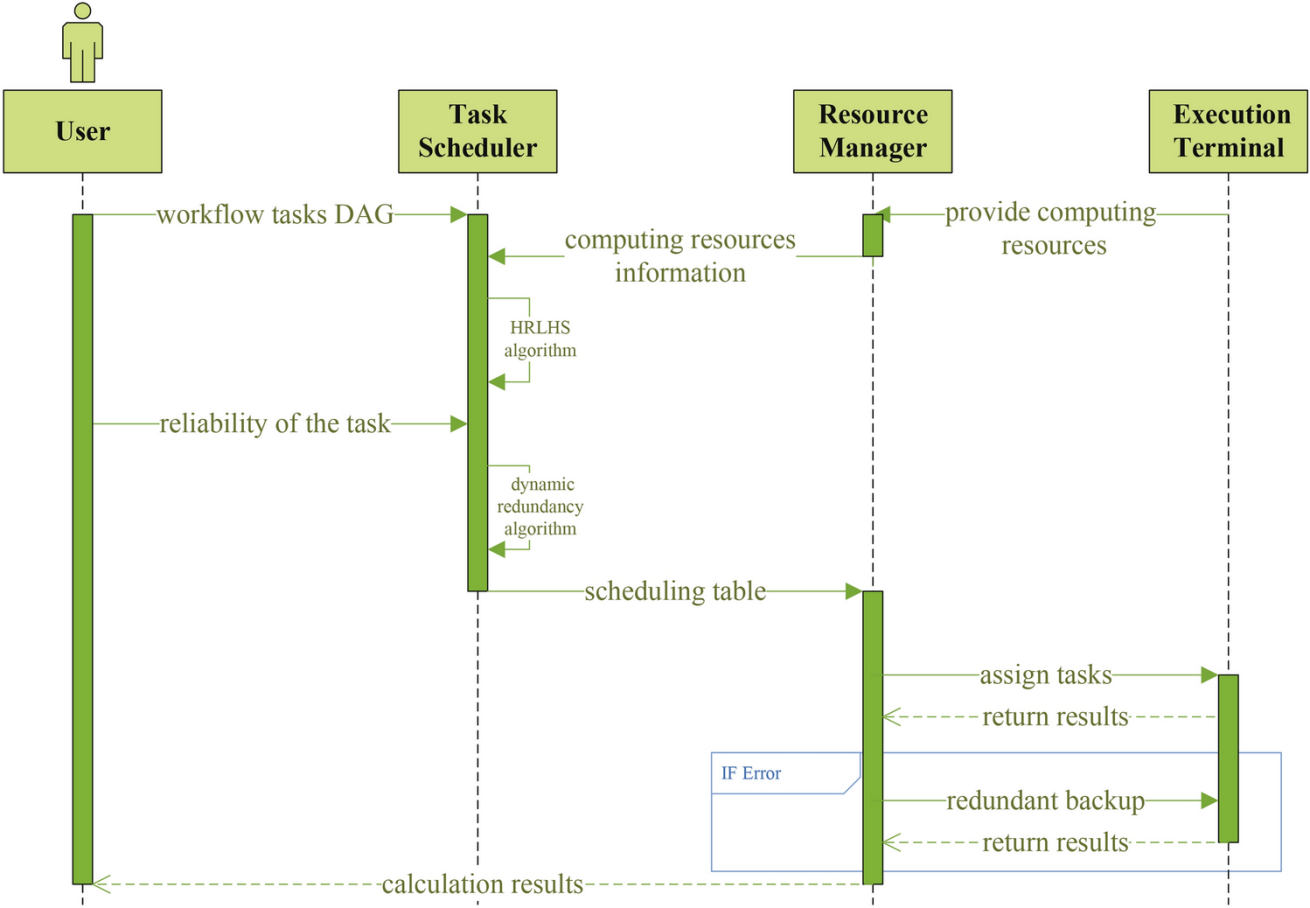
Task scheduling sequence diagram.

In this section, a scheduling algorithm is proposed to address the workflow task scheduling problem for complex computational resources. The algorithm consists of two main phases. In the first phase, a scheduling algorithm named HRLHS is introduced, which ensures energy consumption minimization while meeting the response time requirements. In the second phase, based on the reliability of each task, dynamic redundancy is applied to tasks with lower reliability, ensuring an improvement in the system’s quality of service. To further clarify the interactions between components of the proposed solution, a sequence diagram is provided in Fig. 3 to illustrate the task scheduling processes.

### HRLHS algorithm

Given a DAG as input, the algorithm employs a heuristic approach to explore the initial solution space and utilizes reinforcement learning to adjust the scheduling strategy, balancing exploration and exploitation. This algorithm finds a schedule with the minimum energy consumption while satisfying the response time constraints. The task order must satisfy the following conditions:

14 $$\begin{aligned} & (rank(i) - rank(j)) \times E_{ij} \ge 0 \end{aligned}$$

15 $$\begin{aligned} & flag(j) = tm_{end}^{o(i)} \end{aligned}$$

16 $$\begin{aligned} & tm_{sta}^{o(i)} \ge flag(j) \end{aligned}$$

where *rank*(*i*) represents the priority of task *i*, ensuring that the scheduling of tasks aligns with the logical flow of execution. At the same time, a flag is set at the processor’s completion time for each task. After that, subsequent tasks can start as soon as the flag is activated, ensuring that processor application times do not overlap.

Since edge terminal devices often have limited computational power, storage, and other resources, tasks typically need to be divided into workflows consisting of multiple subtasks, running on different devices^39^. In this context, a DAG is used as input to represent the progressive relationships between tasks and the heterogeneity of computational resources.While NP-hard problems typically use a heuristic algorithm to search for better solutions, such algorithms may lack flexibility when dealing with complex and dynamically changing scenarios. On the other hand, RL algorithms, while highly adaptive, may initially be inefficient and prone to falling into local optima. Therefore, an optimization algorithm called HRLHS, based on a combination of the two approaches, is proposed. Algorithm 1 outlines the details of the proposed HRLHS algorithm. The specific scheduling algorithm is as follows:

**Algorithm 1:**
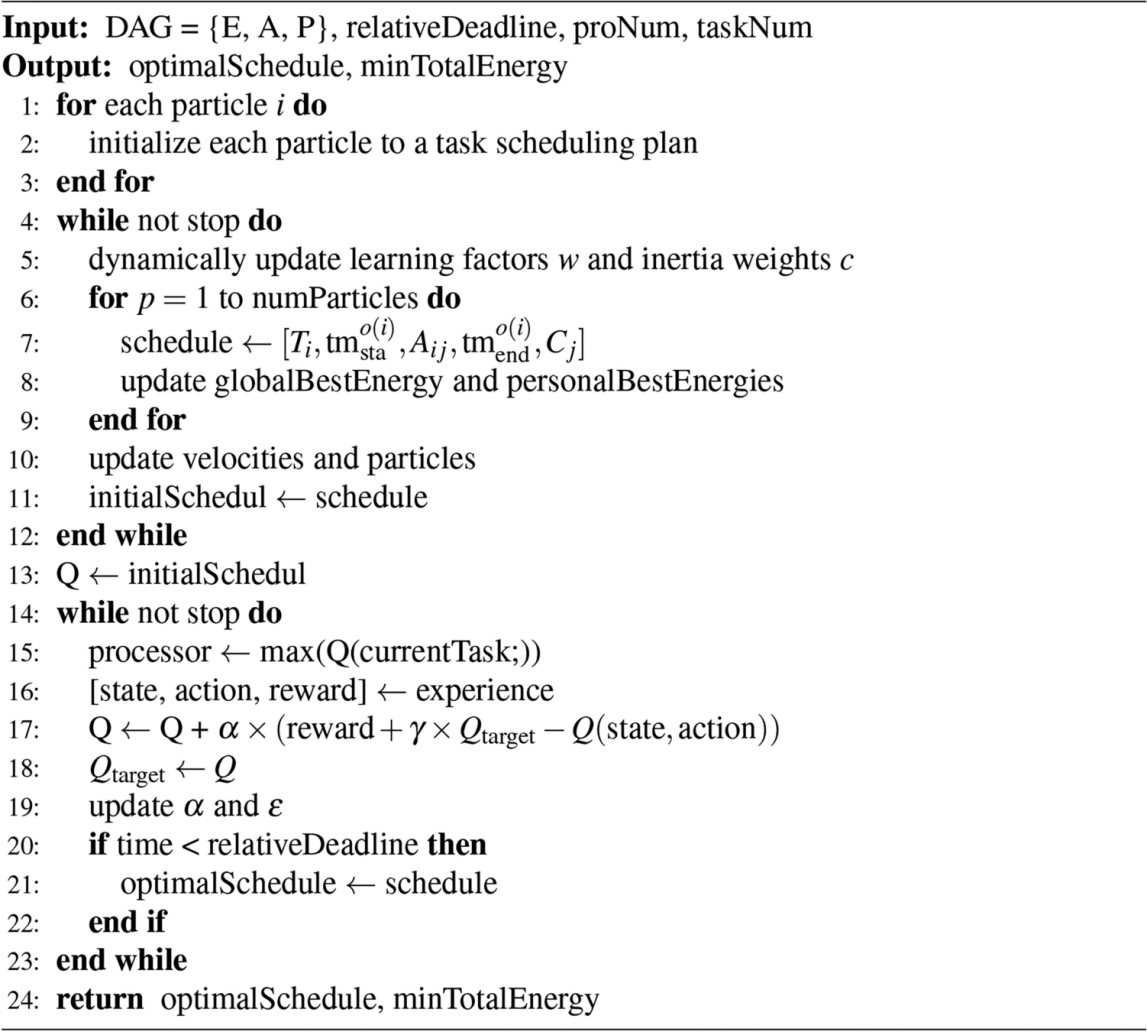
HRLHS

In the initial stage of Algorithm 1, a large inertia weight is assigned to the particles, encouraging exploration of a broader search space for the minimum energy scheduling method, in order to discover potentially optimal solution regions. As iterations progress, the inertia weight is gradually reduced, allowing the algorithm to focus more on the regions with better solutions, thereby improving search efficiency. This strategy helps the algorithm better adapt to complex optimization environments and provides a relatively superior initial solution before reinforcement learning is introduced.

17 $$\begin{aligned} w = w_{\max } - \frac{(w_{\max } - w_{\min })}{T_{\max }} \times t \end{aligned}$$

In this context, *t* represents the current iteration, and $T_{max}$ denotes the maximum number of iterations. The particle’s self-cognition factor and social cognition factor are dynamically adjusted based on the current dispersion of the population. When the population tends toward a local optimum, $c_1$ is increased to enhance the individual exploration ability of the particles; when the population is more dispersed, $c_2$ is increased to promote global learning. Additionally, to prevent the particle swarm from getting stuck in local optima, a detection strategy is employed: if the global optimum does not show significant changes within a certain number of iterations, some particles’ positions or velocities are randomly reset to encourage the algorithm to escape local optima and explore potentially better solutions. Local search and fine-tuning are employed to further refine the candidate solutions in the solution space. For particles that have found relatively good solutions, a local search is performed around them to readjust their solutions. A neighborhood search algorithm is used, where new solutions are randomly generated around the current solution and evaluated for their quality. Moreover, to enhance diversity, perturbations are dynamically triggered when the diversity of the swarm significantly decreases. Perturbations involve making small adjustments to the particles’ positions and velocities to help them escape local traps and readjust their search paths. During the early stages of the search, the algorithm demonstrates strong global exploration capabilities, allowing it to quickly identify good solutions. However, as the iterations progress, the convergence rate gradually slows down.

As an exploration tool in the early stages, the PSO algorithm is capable of quickly searching the solution space and providing an initial solution for reinforcement learning. Its advantage lies in the ability to rapidly traverse a large solution space, thus avoiding the inefficiency of reinforcement learning in the early stages. Building upon this, the reinforcement learning algorithm further refines the initial solution provided by the PSO, focusing on leveraging its adaptability to perform deeper searches based on the existing good solutions. The balance between exploration and exploitation is controlled by the exploration rate, while the learning rate determines the speed and extent of learning.To enhance its efficiency and convergence, a dynamic adjustment mechanism is employed:

18 $$\begin{aligned} & \epsilon = \max (\epsilon _{\text {min}}, \epsilon \times \epsilon _{\text {decay}}) \end{aligned}$$

19 $$\begin{aligned} & \alpha = \max (\alpha _{\text {min}}, \alpha \times \alpha _{\text {decay}}) \end{aligned}$$

**Fig. 4:**
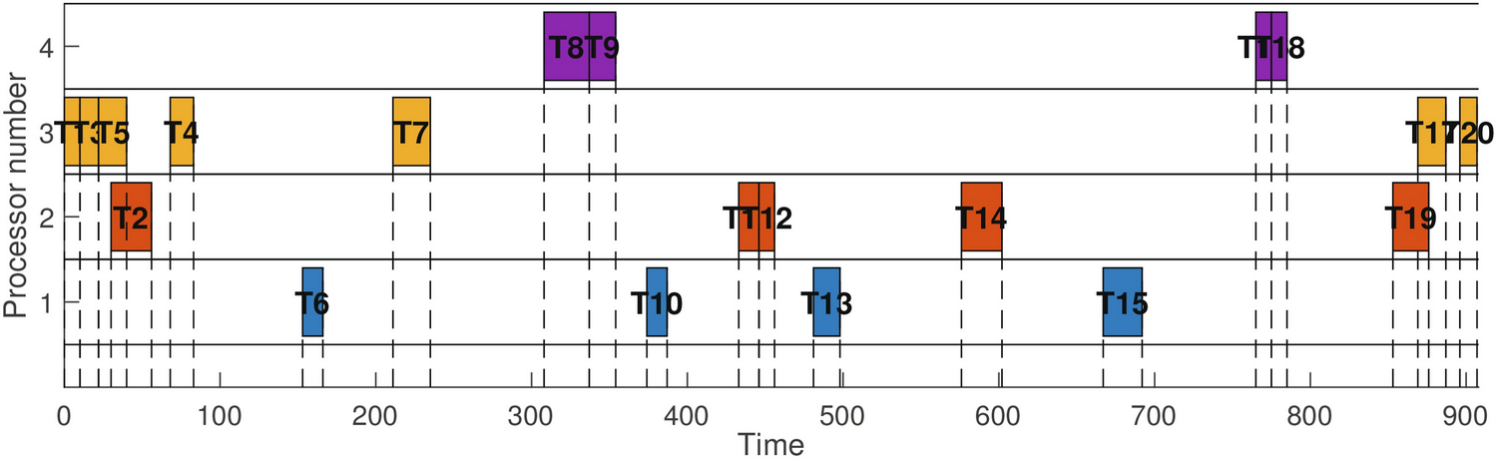
An example of a workflow task scheduling Gantt chart.

The algorithm is designed with parameters for minimum values and decay rates to ensure a transition from more exploration to focused exploitation. To enhance the sample efficiency of reinforcement learning, an experience replay mechanism is adopted. During each iteration, past experiences are stored in a replay buffer and periodically, a batch of samples is randomly drawn from it for training the neural network. This batch processing effectively mitigates the instability in learning caused by the correlation between consecutive samples, thereby improving the stability and efficiency of learning. Subsequently, a double Q-learning mechanism is introduced, maintaining two separate Q-tables to alleviate the overestimation of Q-values. During each update, one Q-value is selected for action selection, while the other is used to update the action value. Additionally, a target network with slower parameter updates is introduced, further enhancing the stability of the algorithm. To optimize the minimum energy consumption scheduling problem, the reward signal is designed to encourage the algorithm to select scheduling methods with lower energy consumption, effectively reducing energy usage. Finally, when neither the reinforcement learning nor the particle swarm optimization algorithm shows significant improvement in solutions, the algorithm terminates and outputs the optimal solution, yielding a scheduling matrix that meets response time requirements with minimal energy consumption, along with illustrative examples such as the scheduling Gantt chart depicted in Fig. 4.

### Dynamic redundancy algorithm

After adopting the HRLHS scheduling algorithm, considering the progressive nature of tasks within a workflow task system, the response time and energy consumption of the overall system will increase in the event of a task failure. To address this issue, a dynamic redundancy strategy is introduced to enhance system reliability. Fault-tolerant scheduling is a key technology in real-time systems, especially in application scenarios with high-reliability requirements, ensuring that tasks are completed correctly within the specified time. The focus of this study is to implement a dynamic proactive redundancy fault-tolerant scheduling mechanism. This mechanism dynamically adjusts during system operation based on the current resource status and potential fault occurrences. Specifically, for critical tasks with lower reliability, the system will perform redundant backup operations. This dynamic adjustment strategy not only improves the system’s adaptability to real-time requirements but also enhances its robustness in uncertain environments. The combination of the scheduling algorithm and dynamic redundancy ensures efficient task execution while reducing the risk associated with task failures. The specific scheduling algorithm is shown as Algorithm 2.

**Algorithm 2:**
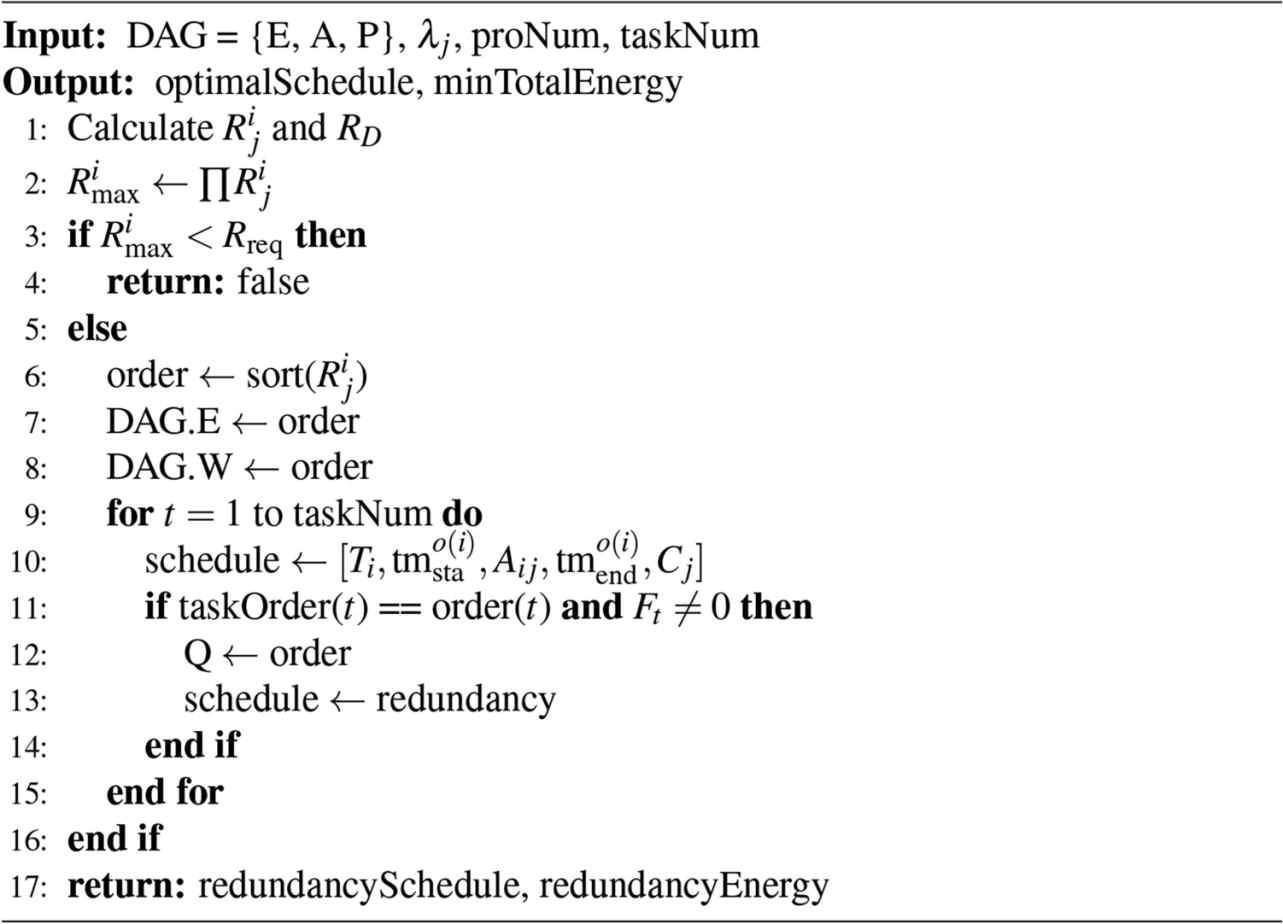
Dynamic redundancy algorithm

First, the reliability of each task running on each processor is calculated based on the reliability parameters of each processor, with the product denoted as the total reliability. This total reliability is then compared with the reliability requirements. If it is higher than the required value, the system can operate; otherwise, the reliability requirements cannot be met. Following the scheduling results, each task is assigned to a unique processor. Tasks with lower reliability scores are selected proportionally for redundant backup processing. It is important to note that the processor selected for the redundant task must not be the same as the original to ensure that in the event of a failure, the backup result data packet can be sent to its subsequent tasks as quickly as possible. This approach of trading space for time enhances the overall quality of service of the system.

When balancing fault tolerance and overhead, the redundancy mechanism of the system must consider both task reliability scores and priorities to achieve an optimal balance between fault tolerance and resource consumption. When a task’s reliability score falls below the system’s required threshold, redundancy backups will be prioritized for these low-reliability tasks to enhance system fault tolerance. However, task priority also plays a critical role in redundancy allocation. For high-priority tasks, even if their reliability score is high, redundancy may still be prioritized because these tasks are crucial to the overall functionality of the system, and their failure could lead to significant performance degradation. Therefore, these tasks may require more redundancy protection. In contrast, for low-priority tasks, despite lower reliability, the system may not allocate excessive redundancy to avoid unnecessary resource consumption. By flexibly adjusting the number of redundancy tasks for low-priority tasks, the system can prioritize resources for more critical tasks, dynamically optimizing redundancy configuration, and ensuring that fault tolerance is improved while maximizing overall system performance and reliability.

### Model time complexity

In Algorithm 1, the time complexity in the first half is mainly concentrated on updating particle information, where each task needs to traverse all other tasks to calculate the precedence dependencies. Let the number of iterations be *I*, and the number of particles be *P*. The time complexity can be expressed as $O(I \times P \times N^2)$. In the reinforcement learning part, each task selects a processor based on the scheduling policy and updates the scheduling information. Let the number of iterations be T, then the time complexity is $O(T \times N \times (M + N))$. Therefore, the total time complexity is $O\left( \max \left( I \times P \times N^2, T \times N \times (M + N) \right) \right)$.

In Algorithm 2, the time complexity can be broken down into several key components. First, calculating the values of $R_i$ and $R_D$ involves traversing all tasks and processors, resulting in a time complexity of $O(N \times M)$. Second, calculating $R_{\text {imax}}$ by multiplying the $R_i$ values across all tasks requires $O(N)$ time. The comparison of $R_{\text {imax}}$ with the required reliability $R_{\text {req}}$ is a constant-time operation, with a complexity of $O(1)$. The sorting of the $R_i$ values to determine the task order takes $O(N \log N)$ time. Then, iterating through all tasks to calculate their scheduling details, such as start and end times, as well as whether redundancy is required, contributes to $O(N)$ time complexity. Finally, scheduling redundant tasks based on the determined order adds another $O(N)$ complexity. Therefore, the total time complexity of Algorithm 2 is $O(N \times M + N \log N + N)$.

In summary, the total time complexity of our algorithms is $O(\max (I \times P \times N^2, T \times N \times (M + N))) + O(N \times M + N \log N + N)$.

## Experiments and discussion

This section provides an explanation of two types of workflows that simulate real-world scenarios and simulate a massive terminal environment. It presents experimental parameters and comparison metrics, analyzing and evaluating the performance of the method proposed in this paper from different perspectives. The experimental environment is set up as Table 3.

**Table 3:** Configuration of the experimental environment.

Experimental environment	Parameter
Operating system	64-bit Windows 11
Processor	AMD Ryzen 5 5600H with Radeon Graphics
CPU frequency	3.3GHz
Memory	32GB RAM
Distributed platform type	Heterogeneous parallel distributed platform
Simulation environment	MATLAB R2023a
Toolbox	MATLAB Basic Toolbox, Optimization Toolbox, Deep Learning Toolbox, Reinforcement Learning Toolbox, etc

### Comparison method

**Fig. 5:**
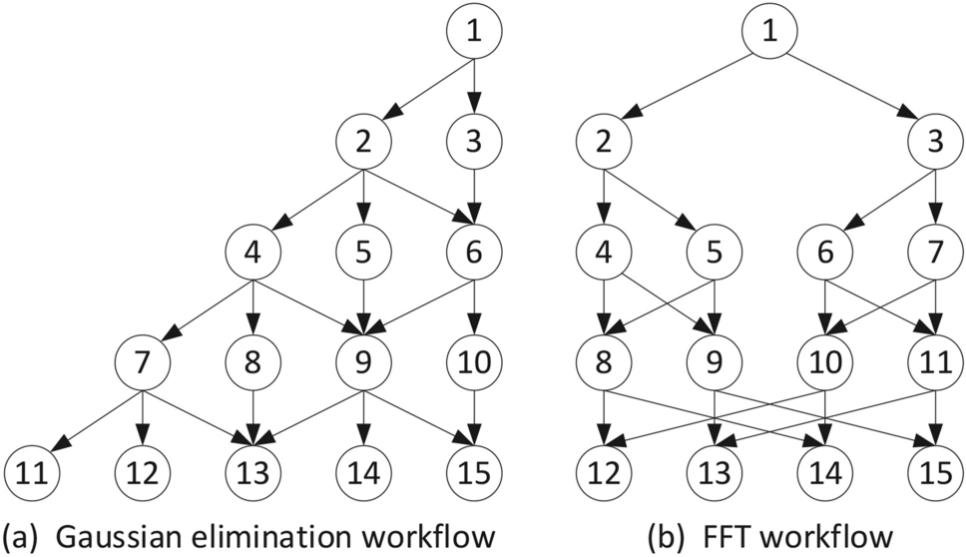
Two real-case parallel workflows.

**Fig. 6:**
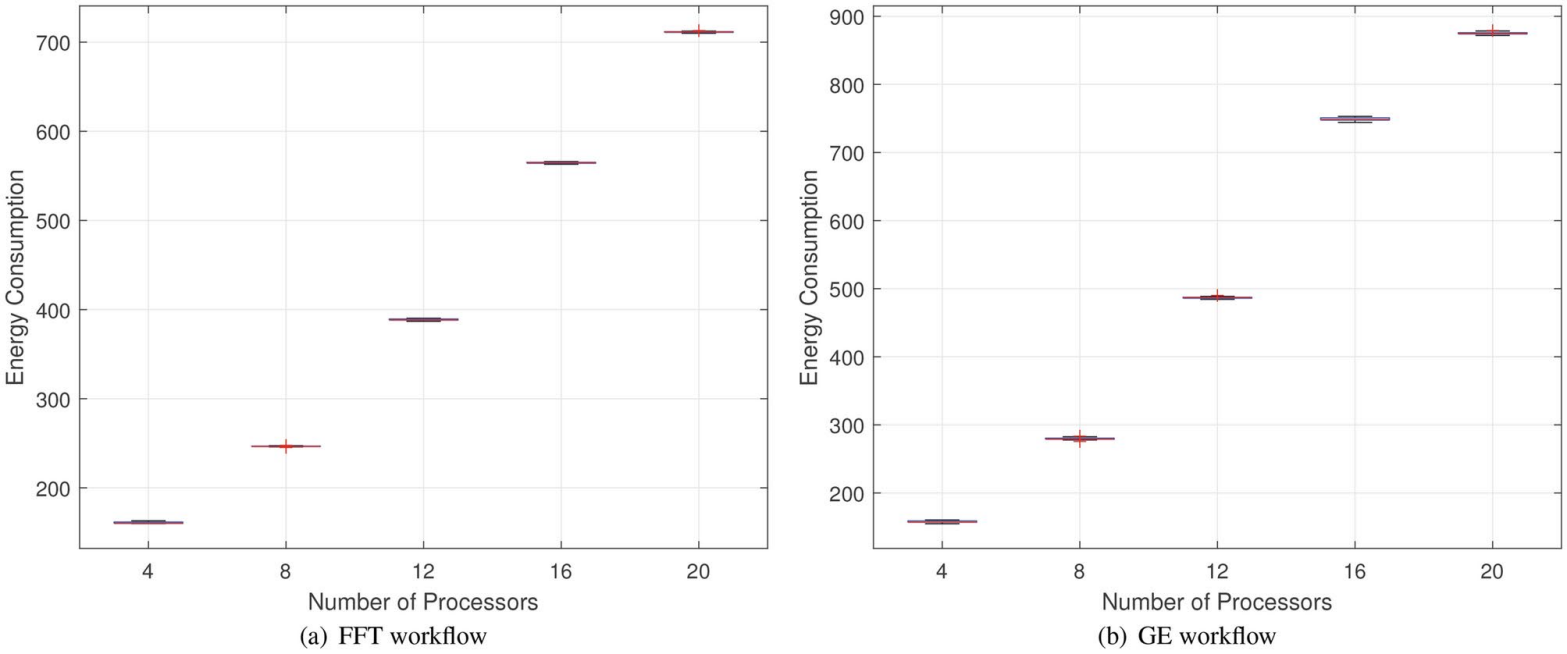
Statistical Analysis of Energy Consumption for two workflows.

This subsection is primarily divided into three parts: comparison with traditional algorithms, comparison with static scheduling, and comparative analysis with the algorithms proposed in papers^19^ and^37^. Among them, the comparison experiment with traditional algorithms employs two benchmark workflows frequently used to evaluate scheduling performance: the Gaussian Elimination (GE) workflow and the Fast Fourier Transform (FFT) workflow, as shown in Fig. 5. The heterogeneous parallelism of the processors is represented by the generation of random parameters. We also conducted a statistical analysis of the evaluation results to ensure the robustness of our findings. Multiple experiments were performed on both workflows, and the corresponding performance data were compiled into box plots, as shown in Fig. 6. These plots offer a clear and intuitive representation of the distribution and variance of the performance metrics across different scenarios. To assess the statistical significance of our proposed method, we employed Analysis of Variance (ANOVA). This method allowed us to compare the means of the performance metrics under different conditions. The results of the hypothesis test indicated that the p-values for all comparisons were below the significance threshold of 0.05, confirming that the observed differences were statistically significant and not due to random chance. This rigorous statistical validation not only strengthens the reliability of our results but also further demonstrates the effectiveness of the proposed method.

**Fig. 7:**
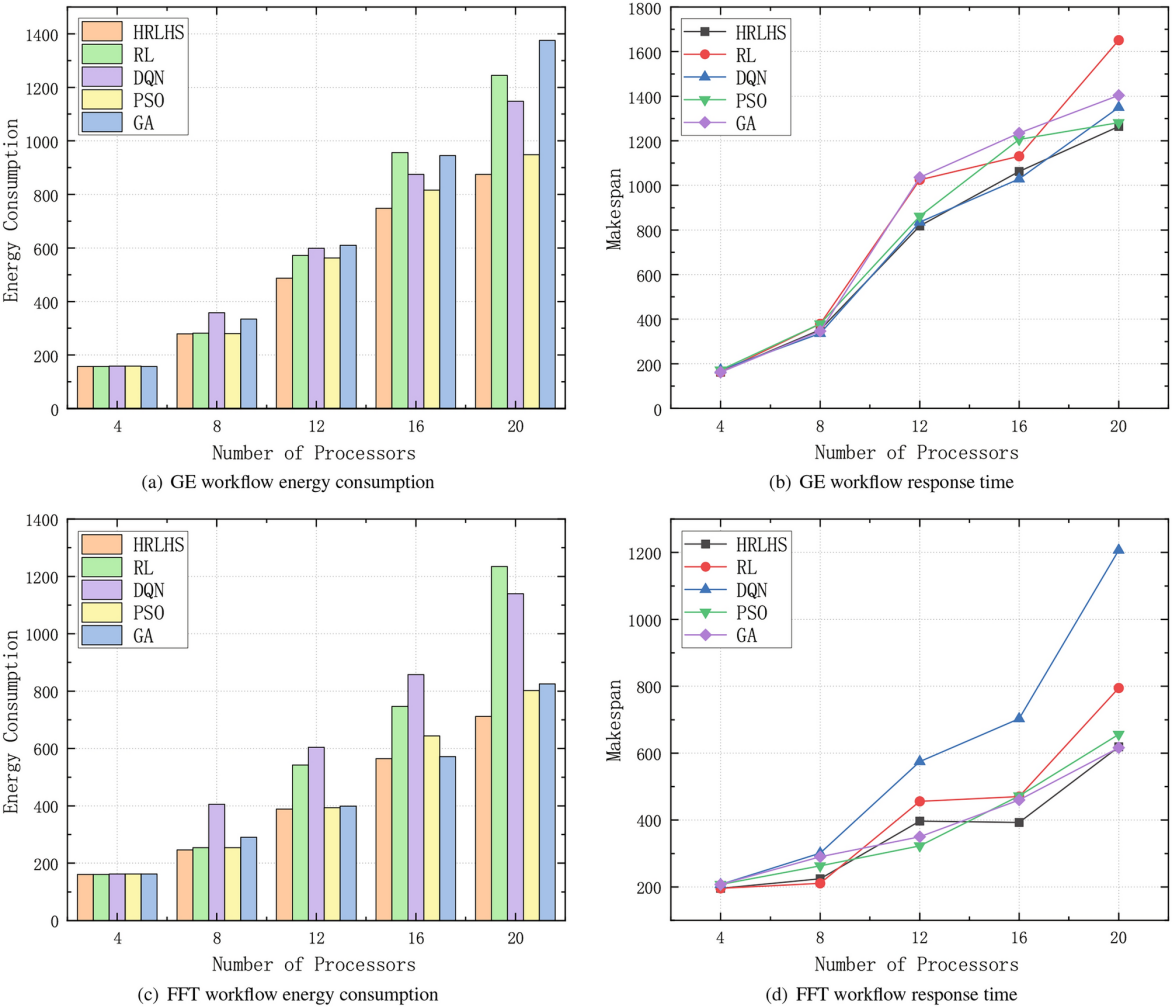
Comparison of energy consumption and response time between the proposed method and the traditional method under the two workflows.

Figure 7 compares the HRLHS algorithm with reinforcement learning, deep reinforcement learning, particle swarm optimization (PSO), and greedy algorithms in terms of energy consumption and response time across two different workflows. The number of heterogeneous parallel processors increases from 4 to 20. It can be observed that as the number of processors increases, the energy consumption and response time of most methods increase linearly, with the HRLHS algorithm consistently maintaining the lowest energy consumption and acceptable response time across multiple scheduling rounds in both workflows. Apart from the proposed HRLHS method, the PSO algorithm performs relatively well, as it can escape local optima and conduct a global search. In contrast, reinforcement learning and deep reinforcement learning tend to place more tasks on the same processor to reduce communication delays, which can lead to higher energy consumption. The greedy algorithm, while prioritizing processors with lower energy consumption for each task, neglects task dependencies, leading to nearly all processors remaining active, which increases overall energy consumption. Moreover, processors that are not used would not contribute to static energy consumption if kept off.

Dynamic scheduling can adjust resources based on real-time system states, whereas static scheduling lacks flexibility and requires pre-planned task assignments. Dynamic scheduling can adapt to sudden changes by reallocating resources according to urgency, priority, task dependencies, and current system status, thereby optimizing either task completion time or energy consumption. This gives dynamic scheduling a distinct advantage.

**Fig. 8:**
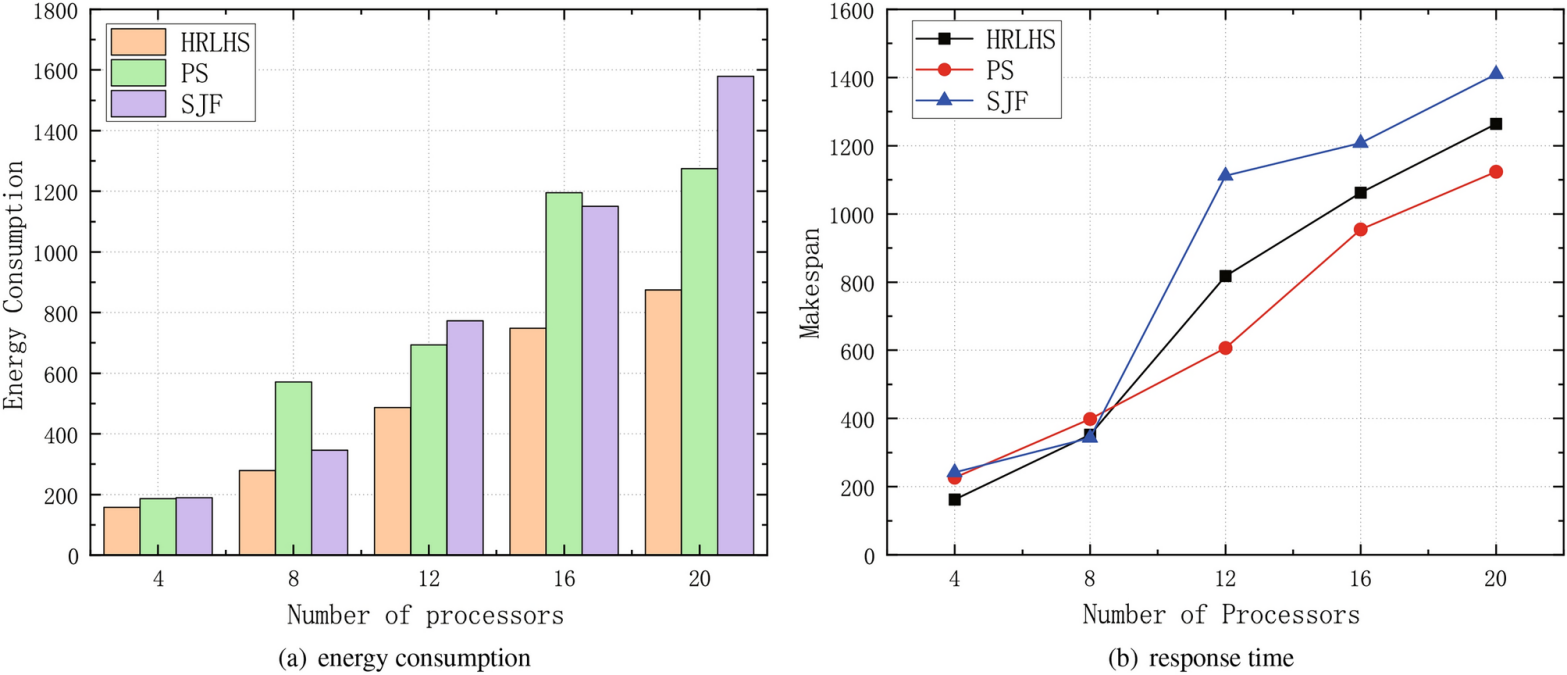
Comparison of energy consumption and latency between dynamic and static scheduling methods.

Figure 8 compares this method with two classic static scheduling algorithms. The Priority Scheduling (PS) method determines the scheduling order based on the energy consumption priority of tasks, while the Shortest Job First (SJF) method reduces dynamic energy consumption by minimizing task execution time. Both algorithms determine the processor selection and task order one by one during scheduling, considering only the dependency relationships between predecessor and successor tasks. They completely ignore the dynamic interactions between workflow tasks, so their scheduling results do not effectively meet the requirements.

**Fig. 9:**
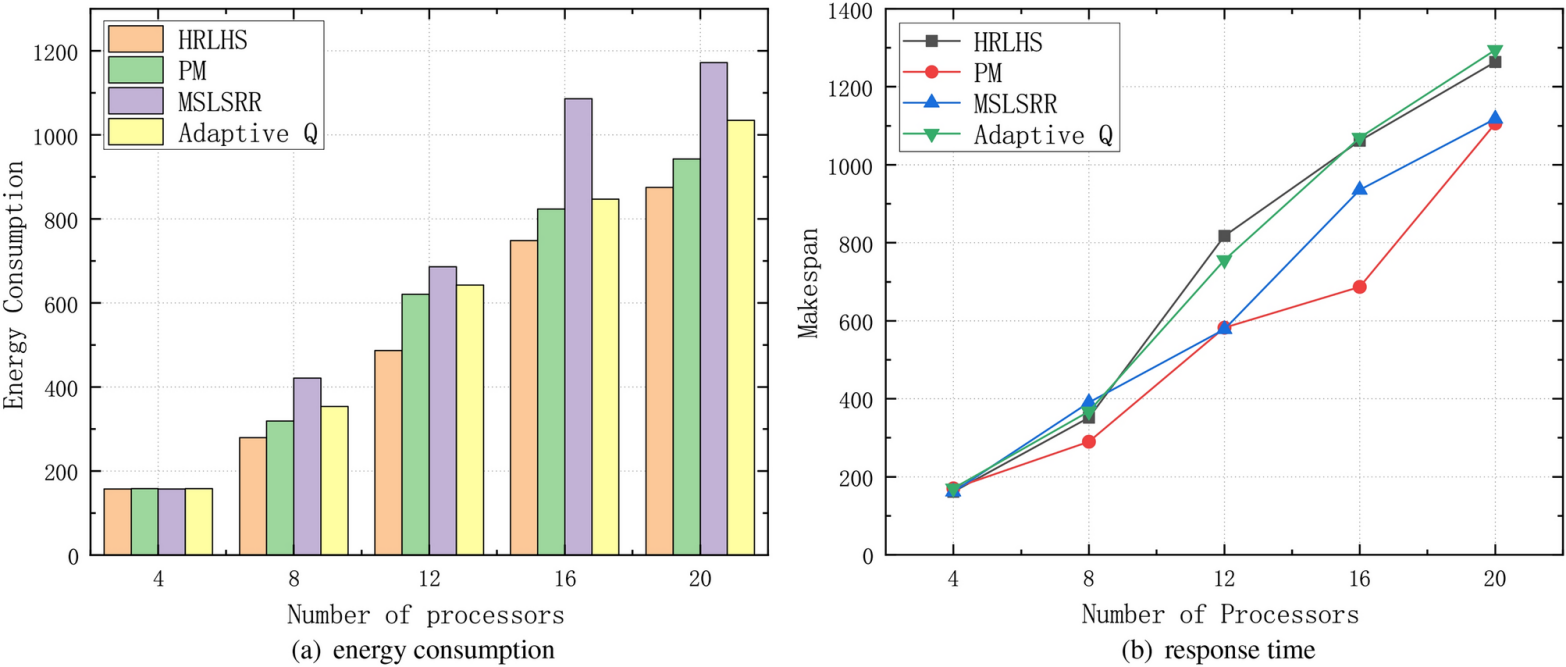
Comparison of energy consumption and latency between our and three advanced methods.

Figure 9 presents the energy consumption comparison between the proposed scheduling algorithm and three existing algorithms: the Processor-Merging (PM) algorithm from paper^19^ and the Minimizing Scheduling Length While Satisfying the Reliability Requirement (MSLSRR) algorithm from paper^37^ and the Adaptive Q-learning algorithm from paper^26^. The experimental results show that the proposed algorithm has significant advantages in energy consumption optimization. The core idea of the PM algorithm is to minimize the number of active processors, thereby reducing the system’s static energy consumption. This is achieved by merging processors to decrease the number of active processors. However, while reducing the number of processors, the PM algorithm overlooks task execution efficiency, leading to suboptimal dynamic energy consumption optimization. The MSLSRR algorithm focuses on minimizing the scheduling length while satisfying system reliability requirements to save energy. It balances task execution time and system reliability during scheduling, aiming to reduce overall energy consumption through effective task scheduling. However, the main focus of this algorithm is on minimizing the scheduling length, with limited optimization of dynamic energy consumption. The Adaptive Q-learning algorithm enables adaptive resource management in complex computing environments but its performance may be influenced by the choice of state and action representations and may require substantial time to converge in highly dynamic environments. In comparison to these algorithms, the proposed algorithm in this paper takes both static and dynamic energy consumption into account and incorporates various optimization strategies during the task-scheduling process. This ensures that energy consumption is minimized while meeting task dependencies and processor load balancing. Experimental results show that the proposed algorithm reduces energy consumption by 14.3% compared to the PM algorithm, by 45.1% compared to the MSLSRR algorithm, and by 26.8% compared to the other algorithm, demonstrating a significant improvement in energy-saving performance.

### Scalability analysis

With the development of the Internet of Things (IoT) and edge computing, an increasing number of terminal devices are simultaneously accessing systems, generating a large volume of task requests and data processing demands. In this context, the system must efficiently manage the resource scheduling, task execution, and energy consumption control of these terminals. Conducting experiments to test the efficiency of the algorithm when handling large-scale tasks is essential. A good scheduling algorithm should maintain stable performance as the task scale increases and avoid performance degradation caused by task backlog or resource bottlenecks.

**Fig. 10:**
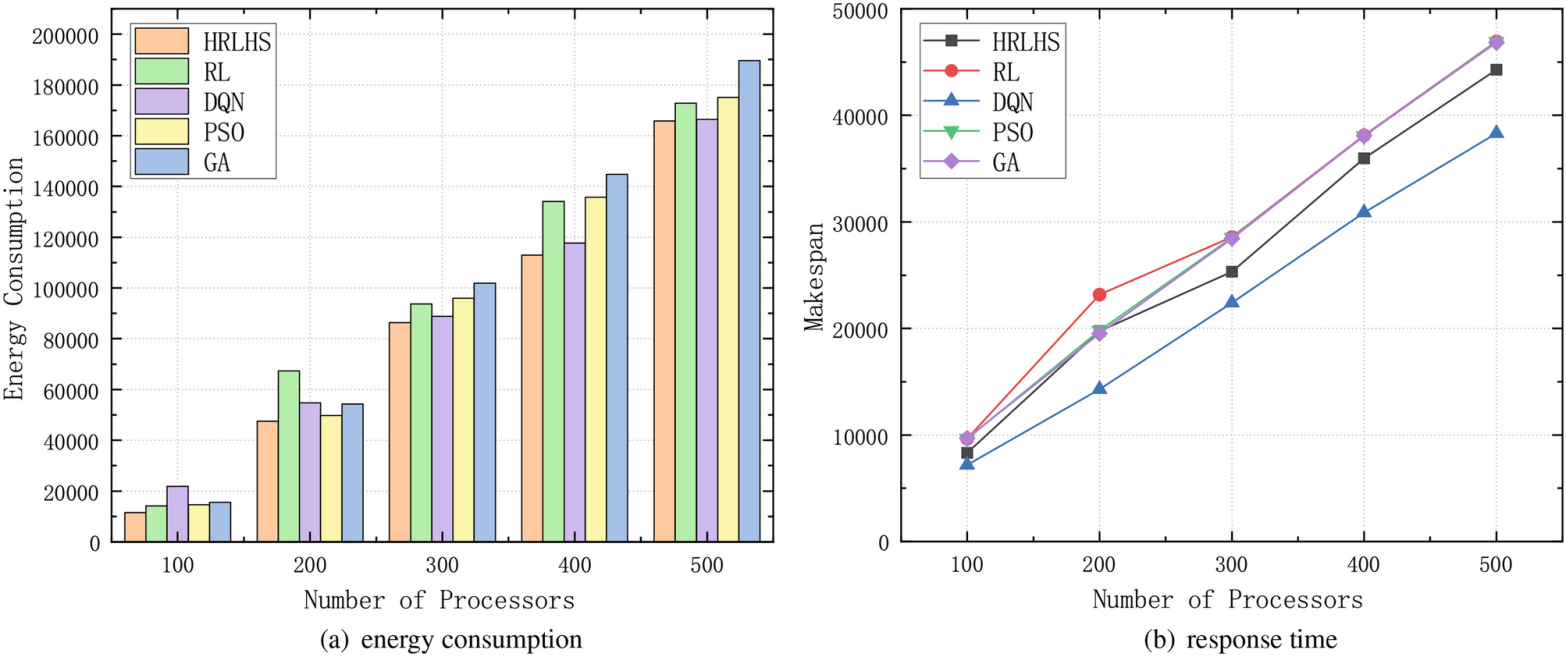
Performance of FFT workflow under massive terminal devices.

**Fig. 11:**
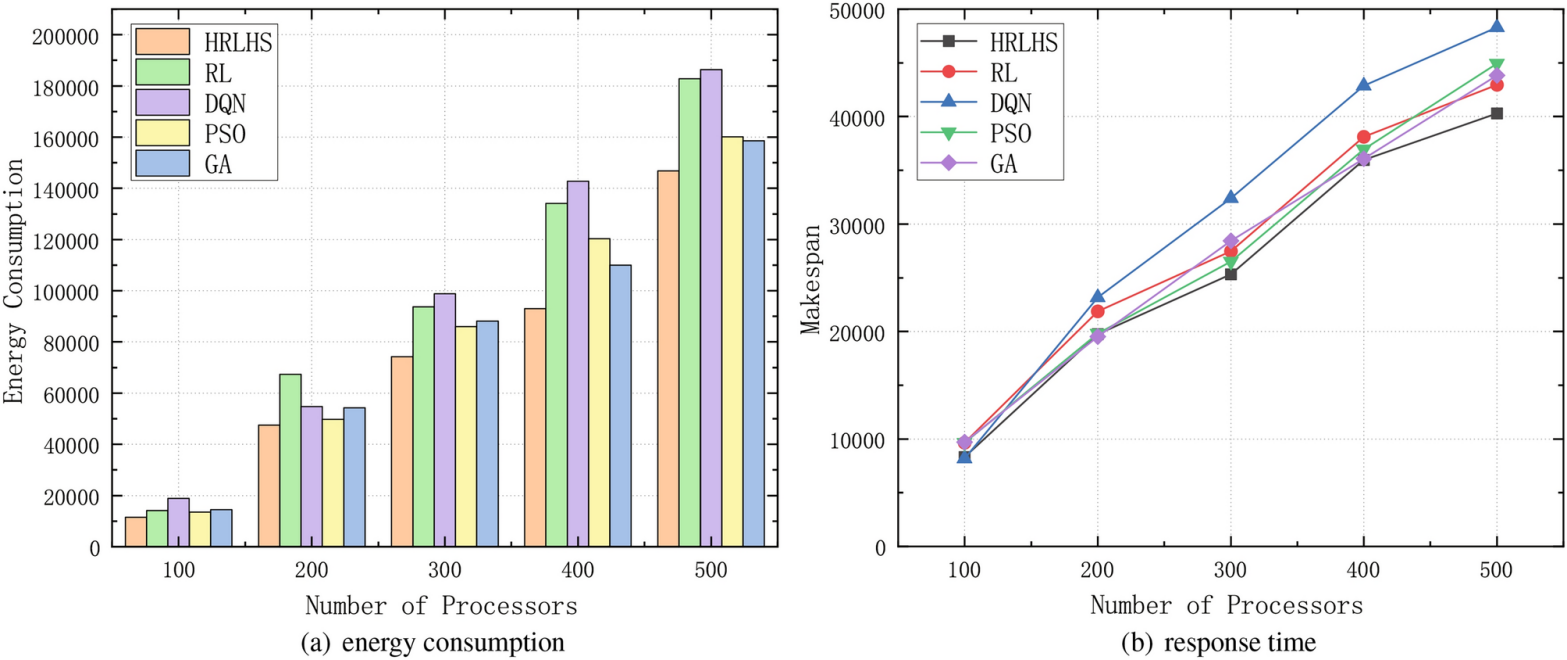
Performance of GE workflow under massive terminal devices.

In order to comprehensively evaluate the scalability of the HRLHS algorithm, we expanded our experimental scenarios to include diverse workloads and edge cases. Specifically, Figs. 10 and 11 illustrate the algorithm’s performance under large-scale data conditions. Figure 10 presents the FFT workflow, a computationally intensive task involving extensive complex number multiplications and additions. It is a suitable benchmark for evaluating the algorithm’s efficiency in handling compute-heavy workloads. Meanwhile, Fig. 11 depicts the GE workflow, which requires frequent broadcasting of pivot rows and synchronization across multiple processors, resulting in a communication-intensive workload. Despite these differing computational characteristics, our proposed method consistently demonstrates strong performance across both scenarios, highlighting its adaptability and effectiveness in diverse workload environments.

In fact, a workflow task is not split into too many parts. A small amount of task splitting can improve the system’s parallel processing capability, thereby enhancing overall efficiency. However, excessive splitting may lead to some negative impacts. First, excessive task splitting increases the scheduling overhead, as more sub-tasks need to be coordinated and managed, resulting in greater consumption of computational resources. Second, splitting tasks into too many parts increases the communication requirements between sub-tasks. Especially in distributed systems, frequent data transmission, and communication delays may become bottlenecks, weakening the advantages of parallel processing. Additionally, the synchronization operations between tasks will become more frequent, causing system waiting time to increase, which in turn prolongs the overall execution time of tasks. Over-splitting may also cause load imbalance, with some processors handling too many tasks while others remain idle, thereby reducing resource utilization efficiency. Therefore, task splitting needs to strike a balance between parallelism and system overhead to avoid increasing scheduling, communication, and synchronization costs, and to achieve the maximum overall efficiency.

### Dynamically redundant scheduling

To enhance the adaptability and efficiency of the scheduling method, the system can incorporate predictive techniques, such as machine learning models, to dynamically adjust the number and allocation of redundant tasks based on processor reliability, task reliability and task failure rates. These models can be trained on historical task performance data, identifying patterns of failure and predicting potential failures. By integrating these predictions into the scheduling process, tasks with higher failure probabilities can be assigned additional redundant tasks, and resources can be allocated more efficiently. For instance, tasks with higher failure likelihoods can be scheduled with more backup tasks, ensuring that failure does not disrupt the overall workflow. These backup tasks are typically identical to the original tasks, performing the same operations to ensure that, in the event of a failure of the original task, the backup task can take over and continue execution. This predictive approach optimizes resource allocation by considering not only the current state of the system but also its historical performance and the potential risks of task failure.

**Fig. 12:**
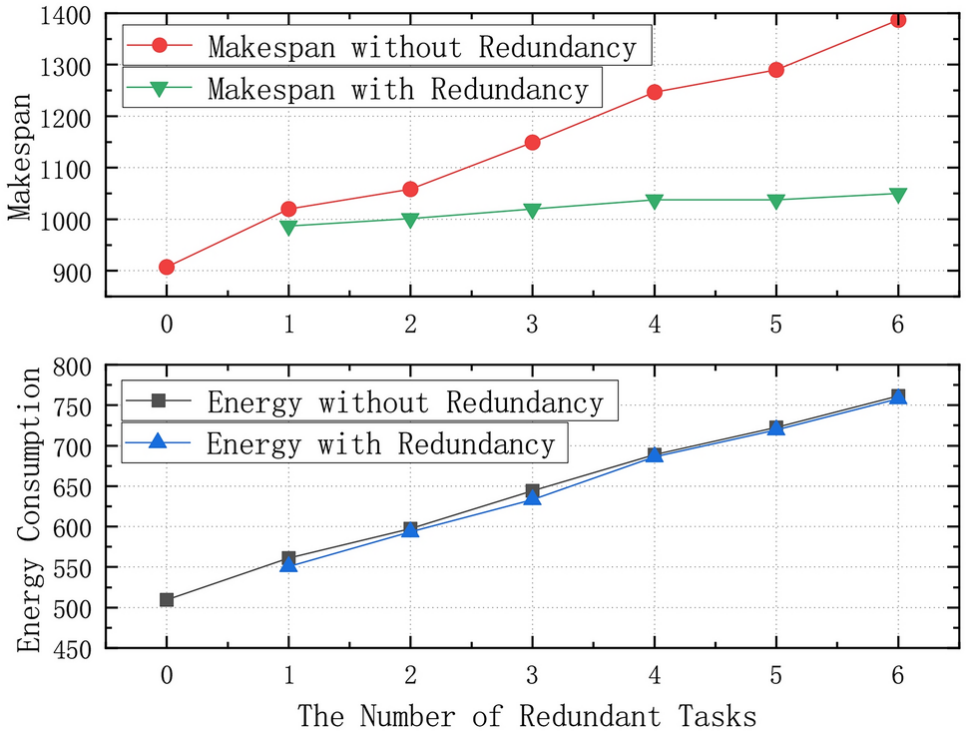
Performance of Dynamic Redundancy Scheduling under Different Numbers of Redundant Tasks.

For a scheduling system, each subtask is an important component of the overall workflow. If an error occurs and recalculation is required, all subsequent tasks must wait, which not only consumes additional time but also increases static energy consumption due to the energy spent on all active processors. Therefore, dynamic redundancy scheduling is adopted. According to the calculation method in formula (7), tasks are first sorted from low to high based on their reliability. Tasks with lower reliability are more likely to fail on the processor. Then, the system checks whether the task has subsequent tasks. For tasks without any subsequent tasks, dynamic redundancy is applied to the few tasks with the lowest reliability, running them simultaneously on the processors with the shortest handling and propagation delays, in addition to the original scheduling results. This way, if a failure occurs in the original scheduling, the data packet can be transferred to the next task with the fastest speed, improving energy efficiency and reducing total response time. Figure 12 shows the performance of dynamic redundancy scheduling for a workflow task system with 12 processors. It compares the ideal energy consumption and completion time of the original workflow with scenarios where 1 to 6 tasks fail, with and without the dynamic redundancy mechanism. The results indicate that implementing dynamic redundancy is indeed necessary.

## Conclusion

For real-world systems, reducing energy consumption has always been one of the key issues and challenges for resource providers. This paper proposes a new algorithm, HRLHS, for dynamic task scheduling of workflows on heterogeneous edge terminals, aiming to reduce energy consumption while ensuring response time requirements. As the tasks in the workflow system exhibit significant progression, a failure at any stage can trigger a chain reaction, impacting the overall system’s response time and energy consumption. To effectively address this challenge, we innovatively introduce a dynamic redundancy strategy to enhance system reliability. The experiments first demonstrate the feasibility of the approach in practical workflows, showing that dynamic scheduling outperforms static scheduling. The paper then compares the proposed algorithm with two existing studies and simulates task scheduling in a massive data environment. The experimental results indicate that the proposed method performs well and achieves excellent results in solving energy consumption issues. In future work, we plan to extend the proposed scheduling algorithm to incorporate distributed computing techniques or cloud-based solutions to address scalability concerns. By leveraging the power of distributed processing frameworks such as Apache Spark or Hadoop, we can handle larger task sets and processor configurations more efficiently. Furthermore, cloud-based solutions can accommodate larger workloads, enabling the system to scale based on workload demands, thereby improving performance and reducing computational bottlenecks.

## Data Availability

Dataset available on request from the authors. The datasets used and analysed during the current study are available from the corresponding author on reasonable request.

## References

[1] Fernando, N., Loke, S. W. & Rahayu, W. Mobile cloud computing: A survey. *Futur. Gener. Comput. Syst.* **29**, 84–106. 10.1016/j.future.2012.05.023 (2013).

[2] Mao, Y., You, C., Zhang, J., Huang, K. & Letaief, K. B. A survey on mobile edge computing: The communication perspective. *IEEE Commun. Surveys Tutorials* **19**, 2322–2358. 10.1109/COMST.2017.2745201 (2017).

[3] Radouane, B., Lyamine, G., Ahmed, K. & Kamel, B. Scalable mobile computing: from cloud computing to mobile edge computing. In: *2022 5th international conference on networking, information systems and security: envisage intelligent systems in 5g//6G-based interconnected digital worlds (NISS)*, 1–6, 10.1109/NISS55057.2022.10085600 (2022).

[4] Phung, T. S., Ward, L., Chard, K. & Thain, D. Not all tasks are created equal: Adaptive resource allocation for heterogeneous tasks in dynamic workflows. In: *2021 IEEE Workshop on Workflows in Support of Large-Scale Science (WORKS)*, 17–24, 10.1109/WORKS54523.2021.00008 (2021).

[5] Beaumont, O., Eyraud-Dubois, L. & Shilova, A. Madpipe: Memory aware dynamic programming algorithm for pipelined model parallelism. In: *2022 IEEE International Parallel and Distributed Processing Symposium Workshops (IPDPSW)*, 1063–1073, 10.1109/IPDPSW55747.2022.00174 (IEEE, 2022).

[6] Raeisi-Varzaneh, M., Dakkak, O., Habbal, A. & Kim, B.-S. Resource scheduling in edge computing: Architecture, taxonomy, open issues and future research directions. *IEEE Access* **11**, 25329–25350. 10.1109/ACCESS.2023.3256522 (2023).

[7] Jiang, J., Sun, Z., Lu, R., Pan, L. & Peng, Z. Real relative encoding genetic algorithm for workflow scheduling in heterogeneous distributed computing systems. *IEEE Trans. Parallel Distrib. Syst.*10.1109/TPDS.2024.3492210 (2024).

[8] Ye, L. et al. Reliability-aware and energy-efficient workflow scheduling in iaas clouds. *IEEE Trans. Autom. Sci. Eng.* **20**, 2156–2169. 10.1109/TASE.2022.3195958 (2022).

[9] Zhao, J., Li, Q., Gong, Y. & Zhang, K. Computation offloading and resource allocation for cloud assisted mobile edge computing in vehicular networks. *IEEE Trans. Veh. Technol.* **68**, 7944–7956. 10.1109/TVT.2019.2917890 (2019).

[10] Amani, N., Pedram, H., Taheri, H. & Parsaeefard, S. Energy-efficient resource allocation in heterogeneous cloud radio access networks via bbu offloading. *IEEE Trans. Veh. Technol.* **68**, 1365–1377. 10.1109/TVT.2018.2882466 (2018).

[11] Ullman, J. D. Np-complete scheduling problems. *J. Comput. Syst. Sci.* **10**, 384–393. 10.1016/S0022-0000(75)80008-0 (1975).

[12] Asghar, H. & Jung, E.-S. A survey on scheduling techniques in the edge cloud: Issues, challenges and future directions. arXiv preprint arXiv:2202.07799 (2022).

[13] Huang, K., Li, R., Gong, W., Wang, R. & Wei, H. Brce: bi-roles co-evolution for energy-efficient distributed heterogeneous permutation flow shop scheduling with flexible machine speed. *Complex Intell. Syst.* **9**, 4805–4816. 10.1007/s40747-023-00984-x (2023).

[14] Zhang, H., Wu, Y. & Sun, Z. Eheft-r: Multi-objective task scheduling scheme in cloud computing. *Complex Intell. Syst.*10.1007/s40747-021-00479-7 (2021).

[15] Liang, Y. *et al.* Distributed and efficient request scheduling in collaborative edge computing. In: *2024 IEEE 44th International Conference on Distributed Computing Systems (ICDCS)*, 1458–1459, 10.1109/ICDCS60910.2024.00150 (2024).

[16] Mishra, P. K. & Chaturvedi, A. K. State-of-the-art and research challenges in task scheduling and resource allocation methods for cloud-fog environment. In: *2023 3rd International Conference on Intelligent Communication and Computational Techniques (ICCT)*, 1–5, 10.1109/ICCT56969.2023.10076030 (2023).

[17] Gohari, P., Voeten, J. & Nasri, M. Work-in-progress: Tight response-time analysis for periodic preemptive tasks under global scheduling. In: *2023 IEEE Real-Time Systems Symposium (RTSS)*, 451–454, 10.1109/RTSS59052.2023.00050 (2023).

[18] Liu, D., Chen, J., Huang, X. & Hong, H. A reliability-aware and energy-aware task scheduling algorithm for heterogeneous multi-core systems. In: *2024 36th Chinese Control and Decision Conference (CCDC)*, 3212–3217, 10.1109/CCDC62350.2024.10587734 (2024).

[19] Hu, B., Cao, Z. & Zhou, M. Energy-minimized scheduling of real-time parallel workflows on heterogeneous distributed computing systems. *IEEE Trans. Serv. Comput.* **15**, 2766–2779. 10.1109/TSC.2021.3054754 (2021).

[20] Meng, S. et al. A fault-tolerant dynamic scheduling method on hierarchical mobile edge cloud computing. *Comput. Intell.* **35**, 577–598. 10.1111/coin.12219 (2019).

[21] Ghorbian, M. & Ghobaei-Arani, M. Function offloading approaches in serverless computing: A survey. *Comput. Electr. Eng.* **120**, 109832. 10.1016/j.compeleceng.2024.109832 (2024).

[22] Ghorbian, M., Ghobaei-Arani, M. & Asadolahpour-Karimi, R. Function placement approaches in serverless computing: A survey. *J. Syst. Architecture*10.1016/j.sysarc.2024.103291 (2024).

[23] Ghorbian, M., Ghobaei-Arani, M. & Esmaeili, L. A survey on the scheduling mechanisms in serverless computing: a taxonomy, challenges, and trends. *Cluster Comput.*10.1007/s10586-023-04264-8 (2024).

[24] Aslani, A. & Ghobaei-Arani, M. Machine learning inference serving models in serverless computing: a survey. *Computing* **107**, 47. 10.1007/s00607-024-01377-9 (2025).

[25] Xu, X., Tian, Q., Xing, Y., Yin, B. & Hu, A. Large-scale data intensive heterogeneous task scheduling method based on parallel gats-ts algorithm. In: *2022 4th International Conference on Communications, Information System and Computer Engineering (CISCE)*, 482–485, 10.1109/CISCE55963.2022.9851157 (2022).

[26] Li, P., Xiao, Y., Yan, J., Li, X. & Wang, X. Reinforcement learning for adaptive resource scheduling in complex system environments. In: *2024 5th International Symposium on Computer Engineering and Intelligent Communications (ISCEIC)*, 92–98, 10.1109/ISCEIC63613.2024.10810157 (2024).

[27] Liao, Z., Peng, J., Xiong, B. & Huang, J. Adaptive offloading in mobile-edge computing for ultra-dense cellular networks based on genetic algorithm. *J. Cloud Comput.* **10**, 1–16. 10.1186/s13677-021-00232-y (2021).

[28] Hu, S. & Li, G. Dynamic request scheduling optimization in mobile edge computing for iot applications. *IEEE Internet Things J.* **7**, 1426–1437. 10.1109/JIOT.2019.2955311 (2019).

[29] Tuli, S., Ilager, S., Ramamohanarao, K. & Buyya, R. Dynamic scheduling for stochastic edge-cloud computing environments using a3c learning and residual recurrent neural networks. *IEEE Trans. Mob. Comput.* **21**, 940–954. 10.1109/TMC.2020.3017079 (2020).

[30] Mahesar, A. R., Xiaoping, L., Sajnani, D. K. & Rajput, K. Y. Efficient workflow scheduling and cost optimization for deadline-constrained microservice applications in mobile edge computing. In: *2024 27th International Conference on Computer Supported Cooperative Work in Design (CSCWD)*, 1931–1936, 10.1109/CSCWD61410.2024.10580475 (2024).

[31] Xia, L. *et al.* Optimal load scheduling based on mobile edge computing technology in 5g dense networking. In: *2022 3rd Asia Conference on Computers and Communications (ACCC)*, 137–142, 10.1109/ACCC58361.2022.00030 (2022).

[32] Dai, H., Liu, S., Liu, B., Fan, Z. & Wang, J. Technical middleware microservice orchestration and fault-tolerant mechanism algorithms for containerized deployment. In: *2024 IEEE 6th International Conference on Civil Aviation Safety and Information Technology (ICCASIT)*, 1611–1616, 10.1109/ICCASIT62299.2024.10828011 (2024).

[33] Long, T., Xia, Y., Ma, Y., Peng, Q. & Zhao, J. A fault-tolerant workflow scheduling method on deep reinforcement learning-based in edge environment. In: *2022 IEEE International Conference on Networking, Sensing and Control (ICNSC)*, 1–6, 10.1109/ICNSC55942.2022.10004189 (2022).

[34] Yao, G., Ren, Q., Li, X., Zhao, S. & Ruiz, R. A hybrid fault-tolerant scheduling for deadline-constrained tasks in cloud systems. *IEEE Trans. Serv. Comput.* **15**, 1371–1384. 10.1109/TSC.2020.2992928 (2020).

[35] Yin, C. & Shi, X. Fault-tolerant scheduling optimization of cloud workflow based on multi-objective optimization algorithm. In: *2023 China Automation Congress (CAC)*, 5916–5921, 10.1109/CAC59555.2023.10450946 (2023).

[36] Chawla, S. & Kaur, A. Fault-tolerant heuristic task scheduling algorithm for efficient resource utilization in cloud computing. In: *2024 International Conference on Automation and Computation (AUTOCOM)*, 131–135, 10.1109/AUTOCOM60220.2024.10486076 (2024).

[37] Xu, H., Zhang, B., Pan, C. & Li, K. Energy-efficient scheduling for parallel applications with reliability and time constraints on heterogeneous distributed systems. *J. Syst. Architect.* **152**, 103173. 10.1016/j.sysarc.2024.103173 (2024).

[38] Liu, Y., Du, C., Chen, J., Du, X. & Yu, W. An energy efficient dynamic scheduling algorithm for reliability constraints in distributed heterogeneous systems. In: *2023 IEEE 11th Joint International Information Technology and Artificial Intelligence Conference (ITAIC)*, vol. 11, 1035–1039, 10.1109/ITAIC58329.2023.10409079 (2023).

[39] Wang, J. et al. Researching the cnn collaborative inference mechanism for heterogeneous edge devices. *Sensors* **24**, 4176. 10.3390/s24134176 (2024). 39000955 10.3390/s24134176 PMC11243860

